# Increased CD127+ and decreased CD57+ T cell expression levels in HIV-infected patients on NRTI-sparing regimens

**DOI:** 10.1186/s12967-017-1367-5

**Published:** 2017-12-20

**Authors:** A. Gonzalez-Serna, S. Ferrando-Martinez, L. Tarancon-Diez, R. S. De Pablo-Bernal, B. Dominguez-Molina, J. L. Jiménez, M. Á. Muñoz-Fernández, M. Leal, E. Ruiz-Mateos

**Affiliations:** 1 Molecular Immunobiology Laboratory, Health Research Institute Gregorio Marañon, Spanish HIV HGM BioBank, Networking Research Center on Bioengineering, Biomaterials and Nanomedicine (CIBER-BBN), General Universitary Hospital Gregorio Marañon, C/Dr. Esquerdo 46, 28007 Madrid, Spain; 2 Viral and Immune Infection Unit Center, Institute of Health Carlos III, Molecular Immunobiology Laboratory, General Universitary Hospital Gregorio Marañon, Majadahonda Campus, Madrid, Spain; 3 Laboratory of Immunovirology, Institute of Biomedicine of Seville (IBiS), Virgen del Rocío University Hospital, C/Avenida Manuel Siurot s/n, 41013 Seville, Spain; 4 Unidad Clínica de Enfermedades Infecciosas y Microbiología, Hospital Universitario Virgen Macarena, Instituto de Biomedicina de Sevilla (IBiS), Universidad de Sevilla, Centro Superior de Investigaciones Científicas, Seville, Spain

**Keywords:** HIV, NRTI-sparing, CD127, CD57

## Abstract

**Background:**

NRTIs-sparing regimens exert favourable profiles on T-cell homeostasis associated parameters. Our aim was to analyze the effect of NRTIs sparing regimen (NRTI-sparing-cART) vs NRTIs-containing regimen (NRTI-cART), on T-cell homeostasis associated parameters in naive HIV-infected patients.

**Methods:**

Biomarkers of cell survival (CD127) and replicative senescence (CD57), were measured by multiparametric flow cytometry for T-cell phenotyping on peripheral blood mononuclear cells (PBMCs) samples just before (baseline) and after 48 weeks of undetectable viral load in patients on NRTI-sparing-cART (N = 13) and NRTI-cART (N = 14). After 48 weeks a subgroup of patients (n = 5) on NRTI-cART switched to NRTI-sparing-cART for another additional 48 weeks. In vitro assays were performed on PBMCs from HIV-uninfected healthy donors exposed or not to HIV. To analyze the independent factors associated with type of cART bivariate and stepwise multivariate analysis were performed after adjusting for basal CD4+, CD8+ and nadir CD4+ T-cell counts.

**Results:**

After 48 weeks of a NRTI-sparing-cART vs NRTI-cART patients have higher effector memory (EM) CD4+ CD127+ T-cell levels, lower EM CD4+ CD57+ T-cell levels, higher CD8+ CD127+ T-cell levels, lower CD8+ CD57+ T-cell levels and higher memory CD8+ T-cell levels. This effect was confirmed in the subgroup of patients who switched to NRTI-sparing-cART. In vitro assays confirmed that the deleterious effect of a NRTIs-containing regimen was due to NRTIs.

**Conclusions:**

The implementation of NRTI-sparing regimens, with a favourable profile in CD127 and CD57 T-cell expression, could benefit cART-patients. These results could have potential implications in a decrease in the number of Non-AIDS events.

**Electronic supplementary material:**

The online version of this article (10.1186/s12967-017-1367-5) contains supplementary material, which is available to authorized users.

## Background

Quality of life and life-length expectancy of HIV-1-infected patients have been significantly improved since the introduction of combination antiretroviral therapy (cART) [1, 2]. However, defects as immune senescence and disturbed T-cell differentiation occur even after long-term suppressive cART [3]. These alterations may underlie the mechanisms causing non-AIDS events, a current major mortality cause in otherwise successfully treated HIV-infected patients [4].

The nucleoside(tide) reverse transcriptase inhibitors (NRTIs) have traditionally been an important back-bone of cART regimens and are currently recommended by all major treatment guidelines [5, 6]. Nonetheless, NRTIs have been associated with immune dysfunction by both short- and long-term exposure [7]. Also, NRTIs have been proposed to accelerate aging by inhibition of telomerase activity [8]. On the other hand, NRTI-sparing regimens seem to exert favourable profiles on T-cell homeostasis-associated parameters in pre-treated and naïve HIV-infected patients [9, 10].

CD57 is a marker of replicative senescence on T cells and cART contributes to decrease its expression on CD8 T cells [11]. Likewise, high CD57 T-cell expression has been associated with HIV-disease progression [12]. Additionally, CD127 expression is crucial for T-cell differentiation and survival through homeostatic proliferation [13]. CD127 expression on T cells is decreased in HIV infection [14], and its down-regulation has been associated with a loss of HIV-specific T-cell function in terms of cytotoxic activity [15], as well as with a failing immune reconstitution under cART [16]. Hence, higher CD127+ and lower CD57+ T-cell expression levels in HIV-infected patients have been associated with lower rates of HIV-disease progression [11–16] but the influence of different types of cART in CD57 and CD127 T-cell expression levels is still unknown.

Thus, our aim was to analyze the effect of a NRTI-sparing regimen on T-cell homeostasis-associated parameters in naive HIV-infected patients, focusing on markers of replicative senescence (CD57) and survival (CD127).

## Methods

### Patients

We retrospectively selected ART-naive HIV-infected patients from a previous study [17], performed between February 2008 and May 2012 at the Virgen del Rocío University Hospital (Seville, Spain), who were initiating: (1) a NRTI-sparing regimen including a CCR5 receptor antagonist (Maraviroc, MVC, 150 mg/24 h) plus a ritonavir-boosted protease inhibitor (PI/rtv, Atazanair) (NRTI-sparing-cART group; n = 13), and (2) standard triple therapy, NRTI-containing regimen (Emtricitabine and Tenofovir [FTC + TDF] or Abacavir and 3TC [ABV + 3TC]) together with either a PI/rtv or a NNRTI (NRTI-cART group; n = 14). Available PBMC samples before cART (baseline) and after 48 weeks of undetectable viral load from patients negative for hepatitis C virus (HCV) antibodies and HCV RNA were collected. After 48 weeks, a subgroup of the NRTI-cART group was simplified to a NRTI-sparing regimen including MVC plus a PI (NRTI-sparing-cART) for an additional 48 weeks (n = 5).

Patients gave written informed consent before entering the study that was approved by the Ethics Committee of the Hospital (Reference Number WS2425049).

### Multiparametric flow cytometry for T-cell immunophenotyping

Thawed PBMCs were stained for extracellular and intracellular flow cytometry. The amine-reactive dye LIVE/DEAD^®^ fixable aqua dead cell stain (Lifetechnologies, Carlsbad, CA) was used to discriminate viable cells. Monoclonal antibodies α-CD3-APC-Cy7, α-CD27-AF700, α-CD127-PECy7 and α-CD57-QD711 were from BD Biosciences (San Jose, CA); α-CD45RA-QD655, α-CD4-QD565 and α-CD8-QD605 were form Life Technologies (Carlsbad, CA). PBMCs were washed and stained with a pretittered quantity of surface antibodies. Cells were then washed and permeabilized using a cytofix/cytoperm kit (BD Biosciences, San Jose CA) according to the manufacturers’ instructions, intracellularly stained α-Bcl-2-APC, washed and fixed in phosphate-buffered saline containing 1% paraformaldehyde (PFA). Flow cytometry was performed on a LSR Fortessa (BD Immunocytometry Systems). A minimum of 3 × 10^5^ events was collected for each condition. Electronic compensation was conducted with antibody capture beads (BD Biosciences, San Jose CA) stained separately with individual monoclonal antibodies. Analysis was performed using FlowJo version 9.2 (Tree Star). T-cell subsets were defined as follows: naive T cells (CD45RA^+^CD27^+^), memory T cells (CD45RO^+^CD27^+^), effector memory T cells (CD45RA^−^CD27^−^), and effector memory RA^+^ T cells (TEMRA) (CD45RA^+^CD27^−^). The accuracy of these phenotypes was previously reported [18].

### In vitro assays

Briefly, PBMCs from healthy donors were isolated on a Ficoll-Hypaque density gradient (Rafer, Spain) following the current procedures of Spanish HIV HGM BioBank [19], stimulated with PHA (1 µg/ml) for 3 days and infected (or not) with HIV for three hours as previously described [20]. After extensive washing, cells were incubated for 3 days with different combination of antiretroviral drugs. The final optimal concentrations used were 20 μM for Abacavir (Kivexa) [8, 21], 5 nM for Darunavir [22] and 10 μM for both, Truvada (Tenofovir plus Emtricitabine) [23–25] and Maraviroc [26]. Finally, multiparametric flow cytometry was performed on these cells, as above mentioned.

### Laboratory tests

Plasma HIV-1 RNA was measured routinely in fresh samples by quantitative PCR (Cobas AmpliPrep/Cobas TaqMan HIV-1 test; Roche Molecular Systems, Basel, Switzerland) according to the manufacturer’s instructions. The lower detection limit was 20 HIV-1 RNA copies/ml. CD4 and CD8 T-cell counts were routinely determined in fresh whole blood using the Epics XLMCL flow cytometer (Beckman-Coulter Inc., California) according to the manufacturer’s instructions.

### Statistical analysis

All continuous variables were expressed as median (interquartile range [IQR]), and categorical variables as numbers and percentage. Spearman test was used to analyze correlations and Wilcoxon for paired samples. Differences between groups were analyzed with the Chi^2^ test and Mann–Whitney U test. A logistic regression was performed to analyze the differences between CD127+ and CD57+ T-cell levels after 48 weeks of follow up adjusting for all variables statistically different between groups at baseline (i.e.: CD4, CD8 and nadir CD4 T-cell counts). Thus, the treatment group was considered the dependent variable and the covariates were CD4+, CD8+, nadir CD4+ T-cell counts and CD127+ or CD57+ T-cell levels. On the other hand, a multivariate linear regression model following a stepwise procedure was used to analyze factors associated CD127+ or CD57+ T-cell levels after 48 weeks of follow up putting together both group of patients. CD127+ or CD57+ T-cell levels were considered the dependent variables and sex, age, time from diagnosis, CD4+, CD8+, nadir CD4+ T-cell counts, viral load and the type of cART were used as covariates. All differences with p < 0.05 were considered statistically significant. Statistical analyses were performed using the Statistical Package for the Social Sciences software (SPSS 22.0; Chicago, IL), and the graphics were generated with Prism, version 5.0 (GraphPad Software, Inc.).

## Results

### Characteristics of the patients

At baseline, those patients who initiated a NRTI-sparing regimen (NRTI-sparing-cART group, n = 13) had higher CD4, nadir CD4 and CD8 T-cell counts compared to patients who initiated a combination of 2 NRTIs and a PI or a NNRTI (NRTI-cART group, n = 14) (Table 1). There were no significant differences in sex, age, time since diagnosis, CD4-T cell percentage, viral load and CD4/CD8 T-cell ratio between both groups. After 48 weeks of suppressive cART there were no differences in CD4 or CD8 levels between both groups (729 [617–887] vs 574 [340–877]; p = 0.202 and 839 [570–1036] vs 693 [460–1053]; p = 0.302, NRTI-sparing-cART and NRTI-cART groups, respectively). After 48 weeks of suppressive cART, a subgroup of patients from the NRTI-cART group (n = 5) was simplified to a NRTI-sparing regimen including MVC plus a PI for an additional 48 weeks. There were no differences in baseline characteristics between this subgroup and the patients who remained in a combination of 2 NRTIs and a PI (data not shown).

**Table 1:** Baseline characteristics of the study subjects (n = 27)

Characteristic	NRTI-sparing-cART (n = 13)	NRTI-cART (n = 14)	*p* value
Sex, male (%)	12 (92.3)	14 (100)	1.000
Age (years)	33 [28–36]	36 [27–48]	0.458
Time since diagnosis (months)	4 [2–31]	3 [2–22]	0.375
CD4+ T-cell count (cells/mm^3^)	474 [421–736]	369 [212–453]	0.007
CD4+ T-cell percentage (%)	28.3 [24.4–32.3]	24.5 [16.0–29.4]	0.094
Nadir CD4+ T-cell count (cells/mm^3^)	402 [322–497]	236 [120–382]	0.017
HIV viral load (log copies RNA/ml)	4.2 [3.7–4.5]	4.7 [4.0–5.2]	0.094
CD8+ T-cell count (cells/mm^3^)	978 [782–1146]	690 [565–828]	0.019
CD4+/CD8+ T-cell ratio	0.6 [0.5–0.7]	0.5 [0.3–0.6]	0.128

### Higher CD127 T-cell levels in patients on a NRTI-sparing-cART

**Fig. 1:**
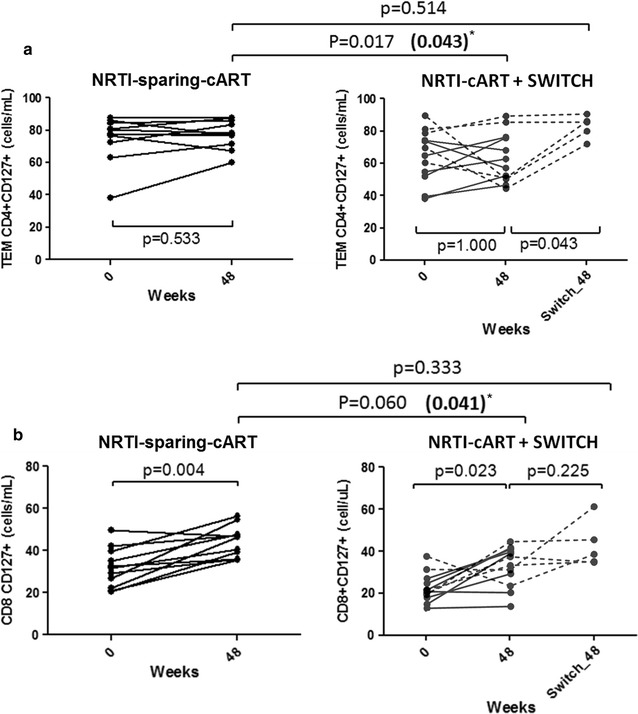
Analysis of CD127^+^ T-cell levels. **a** Higher effector memory (TEM) T CD4+ CD127+ cells levels after 48 weeks of suppressive cART in the NRTI-sparing-cART group compared to the NRTI-cART group. Asterisk, p value in bold letters after adjusting for basal CD4, CD8 and nadir CD4 T-cell counts. The subgroup of the NRTI-cART group who switched to a NRTI-sparing-cART regimen for an additional 48 weeks (n = 5, depicted with dotted lines) significantly increased their TEM CD4+ CD127+ cells to similar levels than the NRTI-sparing-cART group. **b** Higher total CD8+ CD127+ cells levels in the NRTI-sparing-cART group compared to the NRTI-cART group. Asterisk, p value in bold letters after adjusting for basal CD4, CD8 and nadir CD4 T-cell counts. The subgroup of the NRTI-cART group who switched to a NRTI-sparing-cART regimen for an additional 48 weeks (n = 5, depicted with dotted lines) increased their memory T CD8+ CD127+ cells to similar levels than the levels NRTI-sparing-cART group. Variables with a p value of < 0.05 were considered statistically significant

After 48 weeks of suppressive cART, effector memory (TEM) CD4+ CD127+ T-cell levels were higher in the NRTI-sparing-cART group compared to the NRTI-cART group (p = 0.017 in the unadjusted analysis and p = 0.043 after adjusting for basal CD4, CD8 and nadir CD4 T-cell counts to offset the difference found at baseline) (Fig. 1a). Furthermore, the subgroup of the NRTI-cART group who switched to a NRTI-sparing-cART regimen for an additional 48 weeks (n = 5) significantly increased their TEM CD4+ CD127+ cells (p = 0.043) to similar levels than the NRTI-sparing-cART group (p = 0.514). Likewise, total CD8+ CD127+ T-cell levels were higher in the NRTI-sparing-cART group compared to the NRTI-cART group (p = 0.041 after adjusting for basal CD4, CD8 and nadir CD4 T-cell counts) (Fig. 1b). Similarly to TEM CD4+ T cells with CD127 expression, the subgroup of the NRTI-cART group who switched to a NRTI-sparing-cART regimen for an additional 48 weeks (n = 5) reached total CD8+ CD127+ T cells to similar levels than the NRTI-sparing-cART group (p = 0.333).

After 48 weeks of suppressive cART there was a positive correlation between memory CD8 cells and total CD8+ CD127+ in all subjects (r = 0.425; p = 0.034) that did not exist at baseline (r = 0.263; p = 0.226) (Additional file 1: Figure S1a–c). When the patients were grouped according to the cART regimen (including or not NRTIs) this correlation was only found in the NRTI-sparing-cART group (r = 0.555; p = 0.049) (Additional file 1: Figure S1d–f). In addition, bivariate and multivariate analysis confirmed that total CD8+ CD127+ and TEM CD4+ CD127+ T-cell counts were independently associated to the NRTIs absence in the cART regime (Additional file 1: Tables S1, S2).

### Lower CD57 T-cell levels in patients on NRTI-sparing-cART

**Fig. 2:**
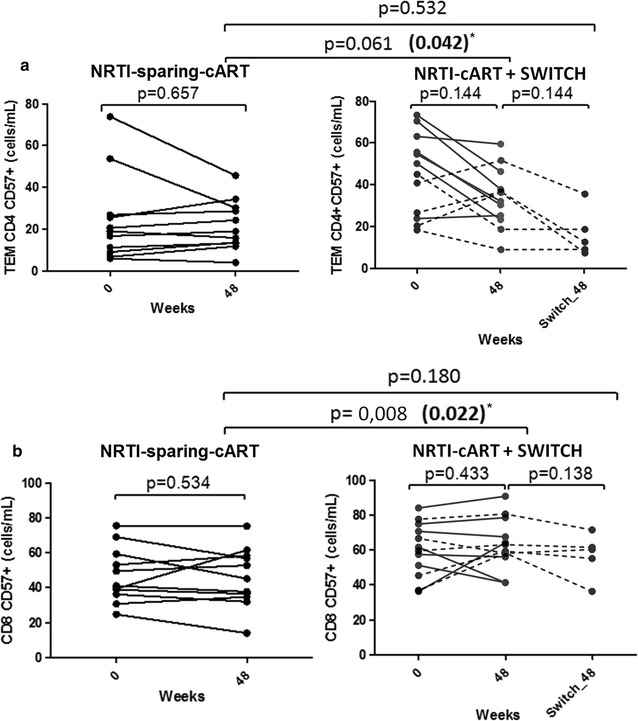
Analysis of CD57+ T-cell levels. **a** Lower effector memory (TEM) T CD4+ CD157+ cells levels after 48 weeks of suppressive cART in the NRTI-sparing-cART group compared to the NRTI-cART group. Asterisk, p value in bold letters after adjusting for basal CD4, CD8 and nadir CD4 T-cell counts. The subgroup of the NRTI-cART group who switched to a NRTI-sparing-cART regimen for an additional 48 weeks (n = 5, depicted with dotted lines) significantly decreased their TEM CD4+ CD57+ cells to similar levels than the NRTI-sparing-cART group. **b** Lower total CD8+ CD577+ cells levels in the NRTI-sparing-cART group compared to the NRTI-cART group. Asterisk, p value in bold letters after adjusting for basal CD4, CD8 and nadir CD4 T-cell counts). The subgroup of the NRTI-cART group who switched to a NRTI-sparing-cART regimen for an additional 48 weeks (n = 5, depicted with dotted lines) decreased their memory T CD8+ CD127+ cells to similar levels than the levels NRTI-sparing-cART group; Variables with a p value of < 0.05 were considered statistically significant

After 48 weeks of antiretroviral treatment, TEM CD4+ CD57+ cell levels were lower in the NRTI-sparing-cART group compared to the NRTI-cART group (p = 0.061 in the unadjusted analysis and p = 0.042 after adjusting for basal CD4, CD8 and nadir CD4 T-cell counts) (Fig. 2a). Similarly to the CD127 expression, the subgroup of the NRTI-cART group who switched to a NRTI-sparing-cART regimen for an additional 48 weeks (n = 5) decreased their TEM CD4+ CD57+ cells to similar levels than the levels NRTI-sparing-cART group (p = 0.532). Likewise, after 48 weeks of suppressive cART, total CD8+ CD57+ T cells levels were lower in the NRTI-sparing-cART group compared to the NRTI-cART group (p = 0.022 after adjusting for basal CD4, CD8 and nadir CD4 T-cell counts) (Fig. 2b). The subgroup of the NRTI-cART group who switched to a NRTI-sparing-cART regimen for an additional 48 weeks (n = 5) decreased their total CD8+ CD57+ T cells to the levels showed by the NRTI-sparing-cART group (p = 0.180).

At baseline, we found a strong negative correlation between memory CD8 cells and total CD8+ CD57+ in all subjects (r = 0.687; p < 0.001). However, after 48 weeks of suppressive cART, this correlation was lost in the NRTI-cART group (r = 0.011; p = 0.974) but it was maintained in the NRTI-sparing-cART group (r = 0.907; p < 0.001) (Additional file 1: Figure S2). Bivariate and multivariate analysis were performed for all patients confirming that total CD8+ CD57+ and TEM CD4+ CD57+ T-cell counts were independently associated to NRTIs containing regimen (Additional file 1: Tables S3, S4).

### Other parameters associated with a better T-cell homeostasis profile in NRTIs-sparing-cART

In order to further analyze the influence of CD127 expression on T-cell homeostasis depending on the type of antiretroviral treatment, we analyze the association of CD127 with the expression of the downstream anti-apoptotic molecule Bcl-2. At baseline we found a strong positive correlation between total CD4+ CD127+ T-cells and CD4+ Bcl2+ T-cells in all patients (r = 0.608; p = 0.001). This correlation was particularly strong in the subsets of TEMRA CD4+ CD127+ and TEMRA CD4+ Bcl2+ cells (r = 543; p = 0.007). After 48 weeks of suppressive cART, this positive correlation was not observed in the NRTI-sparing-cART group (r = 0.271; p = 0.476) but it was maintained in the NRTI-cART group (r = 0.804; p = 0.002) (Additional file 1: Figure S3).

Disturbances in CD127 T-cell expression levels have been associated with the homeostasis of memory CD8 T cells [14]. Analyzing memory CD8 T-cell levels, after 48 weeks of suppressive cART, we found lower memory T CD8 cells levels in the NRTI-cART group compared to the NRTI-sparing-cART group (p = 0.041 after adjusting for basal CD4, CD8 and nadir CD4 T-cell counts) (Additional file 1: Figure S4).

Additionally, after 48 weeks of suppressive cART, we found a trend to lower memory T CD4 cells levels in the NRTI-cART group compared to the NRTI-sparing-cART group (p = 0.072 after adjusting for basal CD4, CD8 and nadir CD4 T-cell counts) (Additional file 1: Figure S5). When the subgroup of the NRTI-cART group switched to a NRTI-sparing-cART regimen for an additional 48 weeks (n = 5) their memory cells significantly increased (p = 0.048) to similar levels than the levels NRTI-sparing-cART group (p = 0.501).

### Effect of different drug combinations in in vitro assays on CD127 and CD57 T-cell levels

**Fig. 3:**
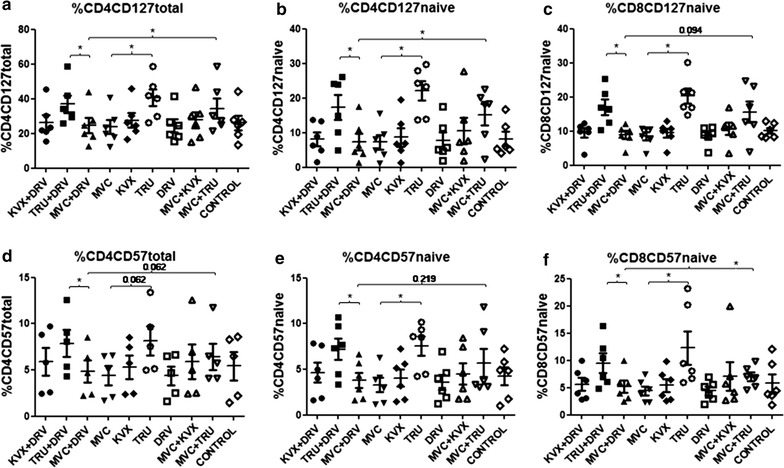
In vitro assays in CD4+ T and CD8+ T cells in the presence of HIV. **a**–**f** In vitro assays confirmed that the deleterious effect of a NRTIs-containing regimen was due to NRTIs, especially the combination tenofovir plus emtricitabine (TRU, Truvada); KVX, Kivexa (combination of Lamivudine plus Abacavir); DRV, Darunavir; MVC, Maraviroc; CONTROL, No drugs; *variables with a p value of < 0.05 were considered statistically significant

The effects of independent or different combination of antiretroviral drugs on CD127 and CD57 T-cell levels were analyzed in vitro. PBMCs from healthy donors were isolated, stimulated with PHA and infected (Fig. 3) or not (Additional file 1: Figure S6) with HIV (n = 6). Then PBMCs were incubated with different combination of antiretroviral drugs. Finally, multiparametric flow cytometry was performed on these cells. In the condition with HIV infection, the highest CD57 levels in both CD4 and CD8 were reached when the combination of Tenofovir plus Emtricitabine was present for all cell subsets but specially for the naive cells, confirming the deleterious effect of a NRTIs-containing regimen (Fig. 3). The same effect was observed for CD127 unlike in vivo. This may be associated with the apoptosis caused by the in vitro conditions which could be counterbalanced by increasing CD127 expression [27]. Results were similar irrespective of the presence of HIV confirming that at least in our system the virus is not affecting the deleterious effect of the drugs (Additional file 1: Figure S6).

## Discussion

Here we show that the implementation of NRTI-sparing regimens (in our study MVC + DRV/rtv) rendered a favourable profile in CD127 and CD57 T-cell expression. These immunomodulatory effects can benefit the immune system integrity of cART-treated patients. This favourable profile was also observed after switching to a NRTI-sparing regimen after being a year on a NRTI-cART containing regimen. Whether the benefits of an NRTI-sparing regimen will be still present in patients with long-term NRTI-containing NRTIs needs further research. Among NRTIs, the combination Tenofovir plus Emtricitabine seems to specially impair this favourable profile.

Reduced expression of CD127 has been associated with progression in HIV infection and bad prognosis of other chronic viral infections as well as in several cancers [28]. This decrease is higher in CD8+ compared to CD4+ T-cell subset [27] what may account for the higher recovery observed in CD8+ CD127+ T-cells in this study in both groups after treatment. Decreased CD127 expression leads to a loss of IL-7 activity contributing to a lower CD8+ cytotoxic T lymphocyte (CTL) activity [28]. In our study, NRTI-cART containing regimens contribute to a lower expression of CD127 expression and NRTI-sparing regimens are able to recover this lower expression after a year on suppressive cART. At baseline we found a strong correlation between the pro-survival factor Bcl-2 and CD127+ in all subjects, specially between TEMRA CD4+ CD127+ and TEMRA CD4+ Bcl2+ cells. This positive correlation was lost in the NRTI-sparing-cART group after 48 weeks of suppressive cART, but it was maintained in the NRTI-cART group. This could be reflecting an attempt to counteract the NRTI-related apoptosis by increasing the expression of antiapoptotic molecules such as Bcl2. These effects seem to be due to the favourable profiles on T-cell homeostasis associated parameters of NRTI-sparing regimens itself or together with some MVC associated immunomodulatory effects [9, 10]. The opposite result regarding CD127 expression in vitro, may reflect the same deleterious effect of NRTIs seen in vivo. In vitro, the increased CD127 expression observed in the Tenofovir plus emtricitabine condition may be associated with increased apoptosis which would be counterbalanced by an increase in CD127 expression. At the long term, the system will be exhausted and CD127 expression may diminish as observed in vivo [28].

CD57 has been described as a marker for replicative senescence on T cells and is associated with a shortening of telomeres after numerous cell divisions [11]. Moreover, NRTIs regimens have been associated with shortening of telomeres [8, 29]. In our study, NRTI-sparing regimens contribute to the downregulation of CD57 expression in HIV infection and are able to reduce the CD57 expression after a year of treatment. At baseline, we found a strong negative correlation between memory CD8 cells and total CD8+ CD57+ in all subjects, but after 48 weeks of suppressive cART this correlation was lost in the NRTI-cART group and maintained in the NRTI-sparing-cART group. Our in vitro assays confirmed that the deleterious effect of a NRTIs-containing regimen was due to NRTIs, especially the combination Tenofovir plus Emtricitabine. Also, the same results were observed irrespective of the presence of the virus. This would confirm that, at least in our system, the drugs rather than the HIV infection are the main drive of this deleterious phenotype. These results could have relevance in pre-exposure prophylaxis strategies. On the other hand, the presence of NRTIs could favor the persistence of cells in replicative senescence phase expressing CD57. A decrease of CD57 due to NRTI-sparing regimens would lead to a reduction of the HIV ability to persist in cells resistant to apoptosis, and therefore to a decrease of the viral reservoir, but more robust studies would be needed to confirm this hypothesis. The clinical relevance of CD57 expression in HIV infection is still not well understood and our data contribute to a better understanding of this issue. All together, our data support the idea that memory T-cell differentiation and the premature ‘ageing’ of the immune system, can be improved after successful viral suppression with NRTI-sparing regimens.

Our study has several limitations. It is an observational study, and our results were obtained retrospectively. A randomized controlled trial would be more definitive in proving that NRTI-sparing regimens are a more beneficial strategy. In our study, patients who initiated a NRTI-sparing regimens had higher CD4 levels, nadir CD4 and CD8 levels compared to those who initiated a combination of 2 NRTIs and a PI. However, our main results were obtained after adjusting for basal CD4, CD8 and nadir CD4 T-cell counts in order to minimize this limitation. Also, as an intragroup control, the subgroup of patients who switched to a NRTI-sparing regimen after being a year on a NRTI-cART containing regimen tended to recover the favourable profile of these markers. In addition, although MVC is underutilized as compared to integrase strand transfer inhibitors and NRTI-sparing two-drugs regimens including MVC were reported to be inferior to NRTIs-based three-drugs regimens in antiretroviral-naive adults [30], regimens including MVC has been associated to a better immunomodulatory profile as improve duodenal immunity and less bone loss, among others, in several clinical trials also in antiretroviral-naive adults [31–33]. Moreover, we cannot know whether the effects observed in vivo are because of the NRTI-sparing regimen, MVC or both but based on our results in vitro we think that NRTI-sparing regimen is playing a main role in this matter. Finally, the effects observed in vitro with the combination Tenofovir plus Emtricitabine could be due to the documented side effects of Tenofovir disoproxil fumarate [34]. A new drug, Tenofovir alafenamide, has been recently introduced and also administered plus Emtricitabine [35]. This new drug claims to avoid most of the previous described side effects of Tenofovir disoproxil fumarate [36, 37]. Therefore, maybe our in vitro results obtained using Tenofovir disoproxil fumarate could change using this new combination with Tenofovir alafenamide instead.

## Conclusions

Understanding how CD127 and CD57 are regulated during HIV infection will provide insight due to the favorable profiles on T-cell homeostasis associated parameters or the development of novel therapeutics to improve immune function and anti-viral T-cell activity. On the basis of these results we think that the implementation of NRTI-sparing regimens, with a favourable profile in CD127 and CD57 T-cell expression, could benefit cART-patients thanks to these immunomodulatory effects and this could involve a decrease in the number of non-AIDS events [17].

## Additional file

**Additional file 1.** Additional figures and tables.
