# Supplementary material for: Increased CD127+ and decreased CD57+ T cell expression levels in HIV-infected patients on NRTI-sparing regimens
Source: J Transl Med. 2017 Dec 20;15:259. doi: 10.1186/s12967-017-1367-5 (PMC5738860; doi:10.1186/s12967-017-1367-5)
Supplement: Supplementary file 1 — Additional file 1. Additional figures and tables. [file 12967_2017_1367_MOESM1_ESM.doc]

**Additional material:**

**Figure S1:**


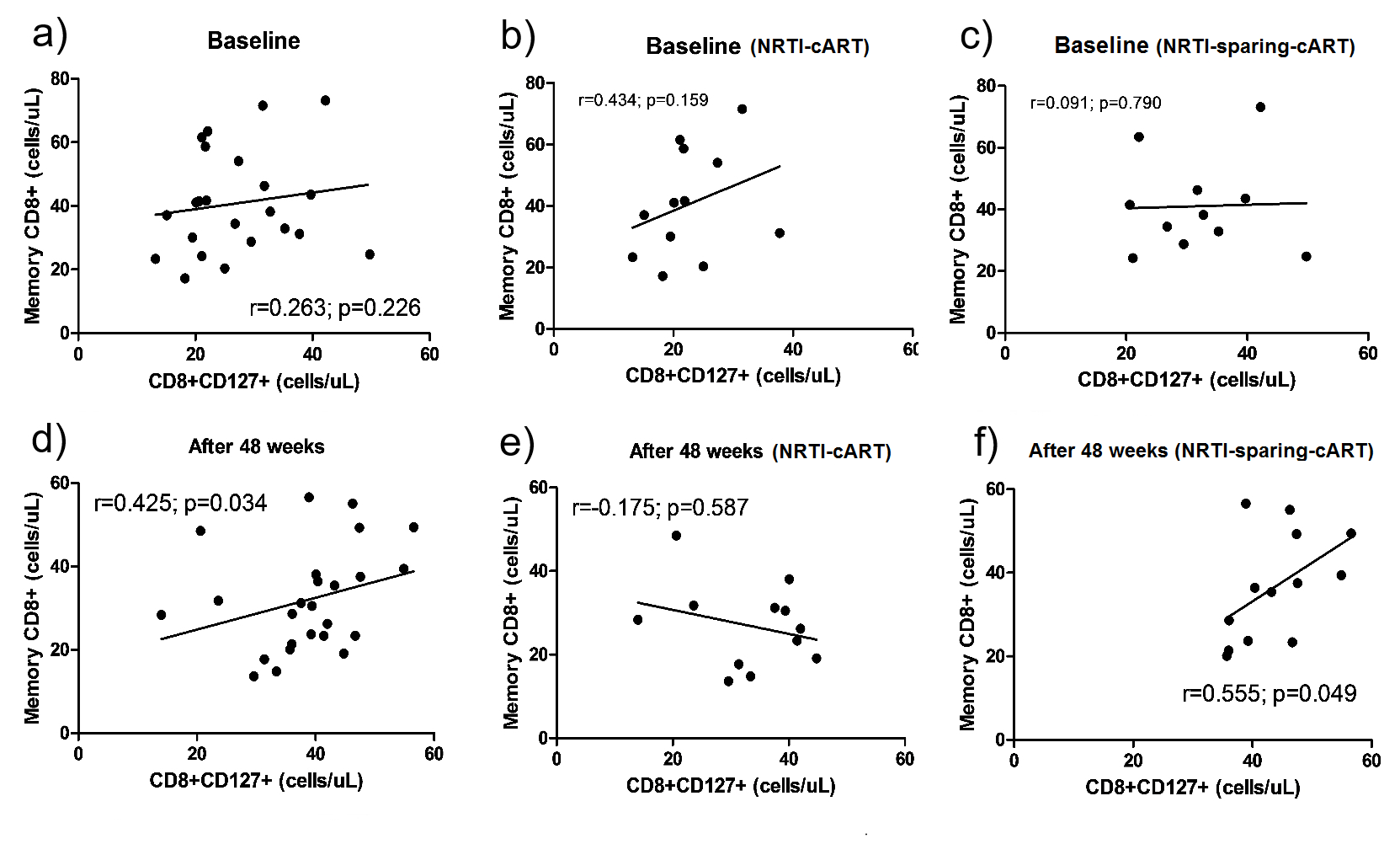


**a-f)** Positive correlation between memory CD8+ cells and CD8+CD127+ in both groups after 48 weeks of suppressive cART but not at baseline. This correlation was mainly due to the association between memory CD8+ cells and CD8+CD127+ in NRTI-sparing-cART group. Variables with a p-value of <0.05 were considered statistically significant.

**Figure S2:**


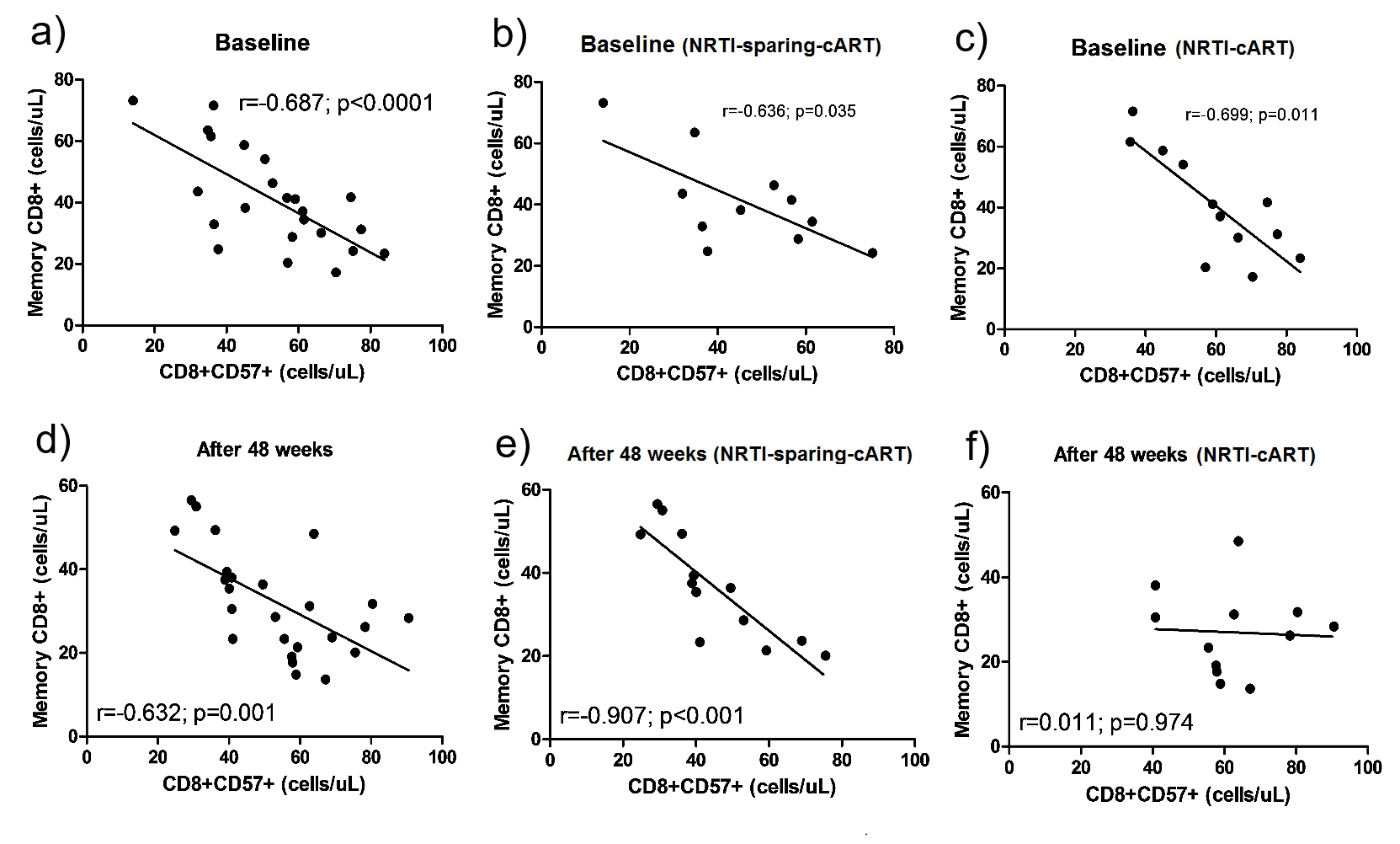


**a-f)** Negative correlation between memory CD8+ cells and CD8+CD57+ in both groups at baseline. This correlation was lost after 48 weeks of suppressive cART only in the NRTI-cART group. Variables with a p-value of <0.05 were considered statistically significant.

**Figure S3:**


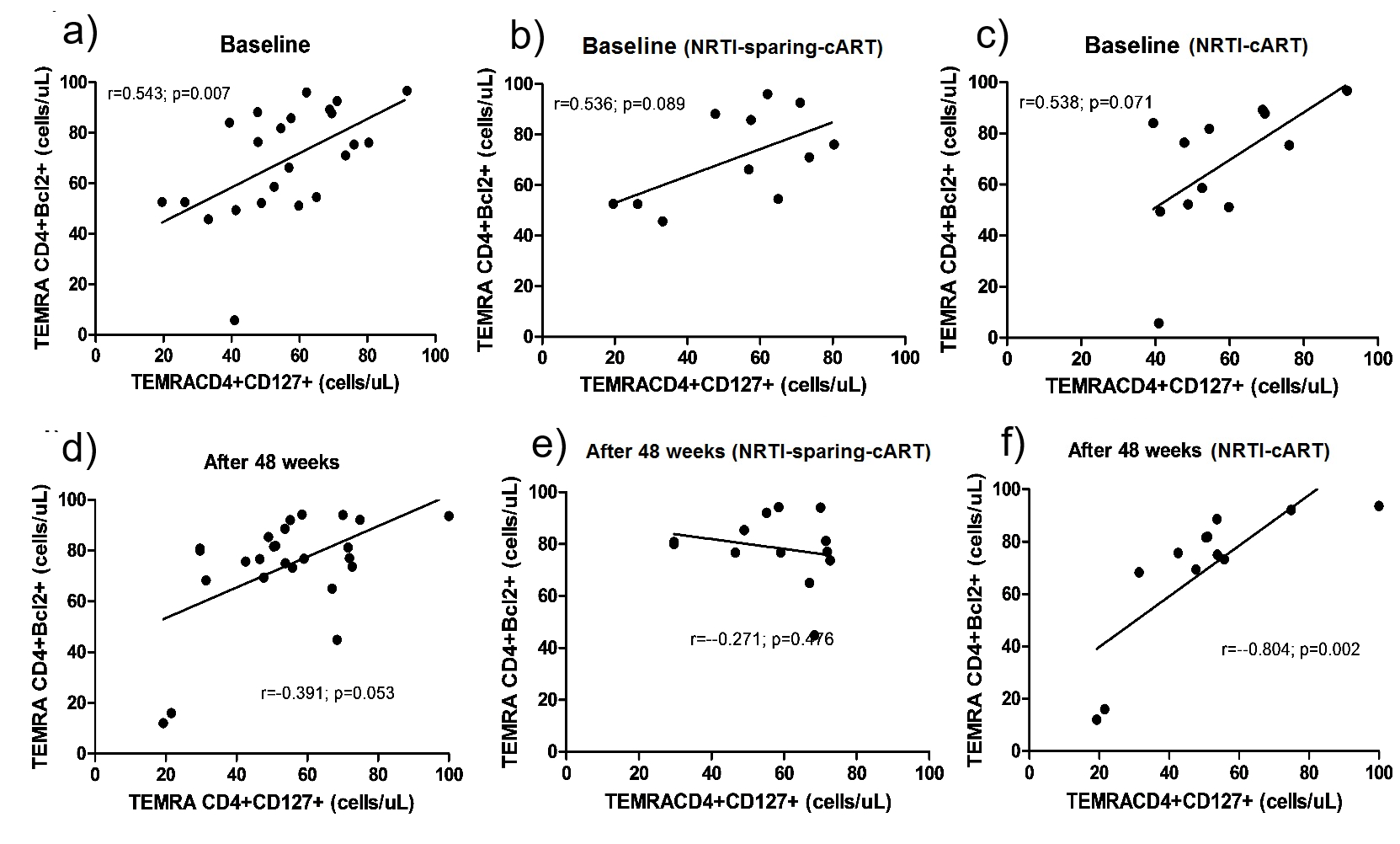


**a-f)** Strong positive correlation between CD4+CD127+ cells and antiapoptotic CD4+Bcl2+ cells in all patients (r=0.608; p=0.001). This correlation was mainly due to correlation between TEMRA CD4+CD127+ and TEMRA CD4+Bcl2+ cells. After 48 weeks of suppressive cART, this positive correlation was lost only in the NRTI-sparing-cART group. Variables with a p-value of <0.05 were considered statistically significant.

**Figure S4:**

**
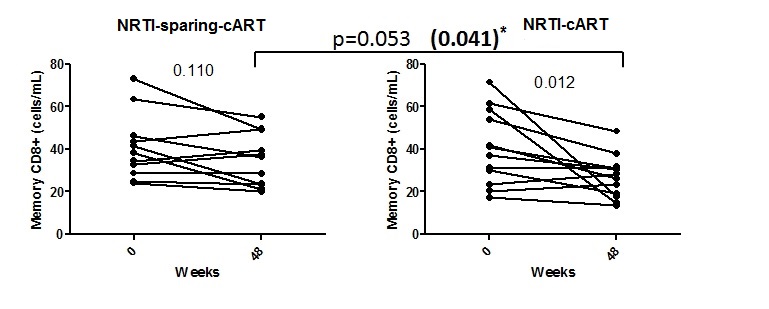
**

Lower memory T CD8 cells levels in the NRTI-cART group compared to the NRTI-sparing-cART group after 48 weeks of suppressive cART. *****, p-value in bold letters after adjusting for basal CD4, CD8 and nadir CD4 T-cell counts. Variables with a p-value of <0.05 were considered statistically significant.

**Figure S5:**

**
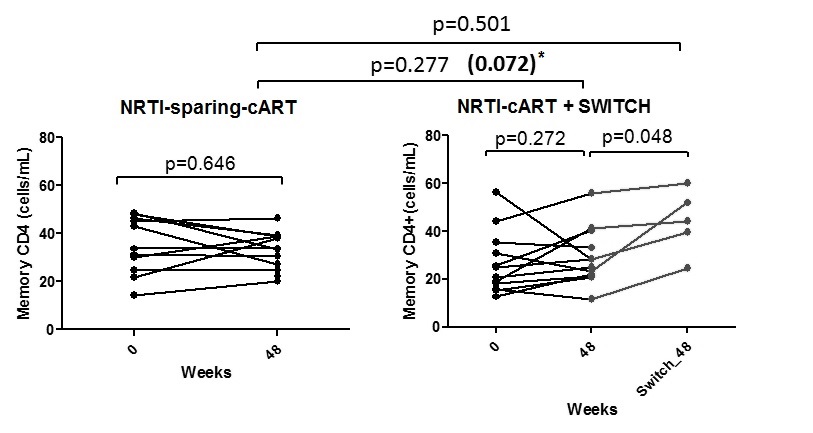
**

Trend to lower memory T CD4 cells levels in the NRTI-cART group compared to the NRTI-sparing-cART group group (*****, p-value in bold letters after adjusting for basal CD4, CD8 and nadir CD4 T-cell counts) after 48 weeks of suppressive cART. When the subgroup of the NRTI-cART group switched to a NRTI-sparing-cART regimen for another 48 additional weeks their memory cells significantly increased to similar levels than the levels NRTI-sparing-cART group. Variables with a p-value of <0.05 were considered statistically significant.

**Figure S6**. In vitro assays in CD4+T and CD8+T cells without the presence of HIV


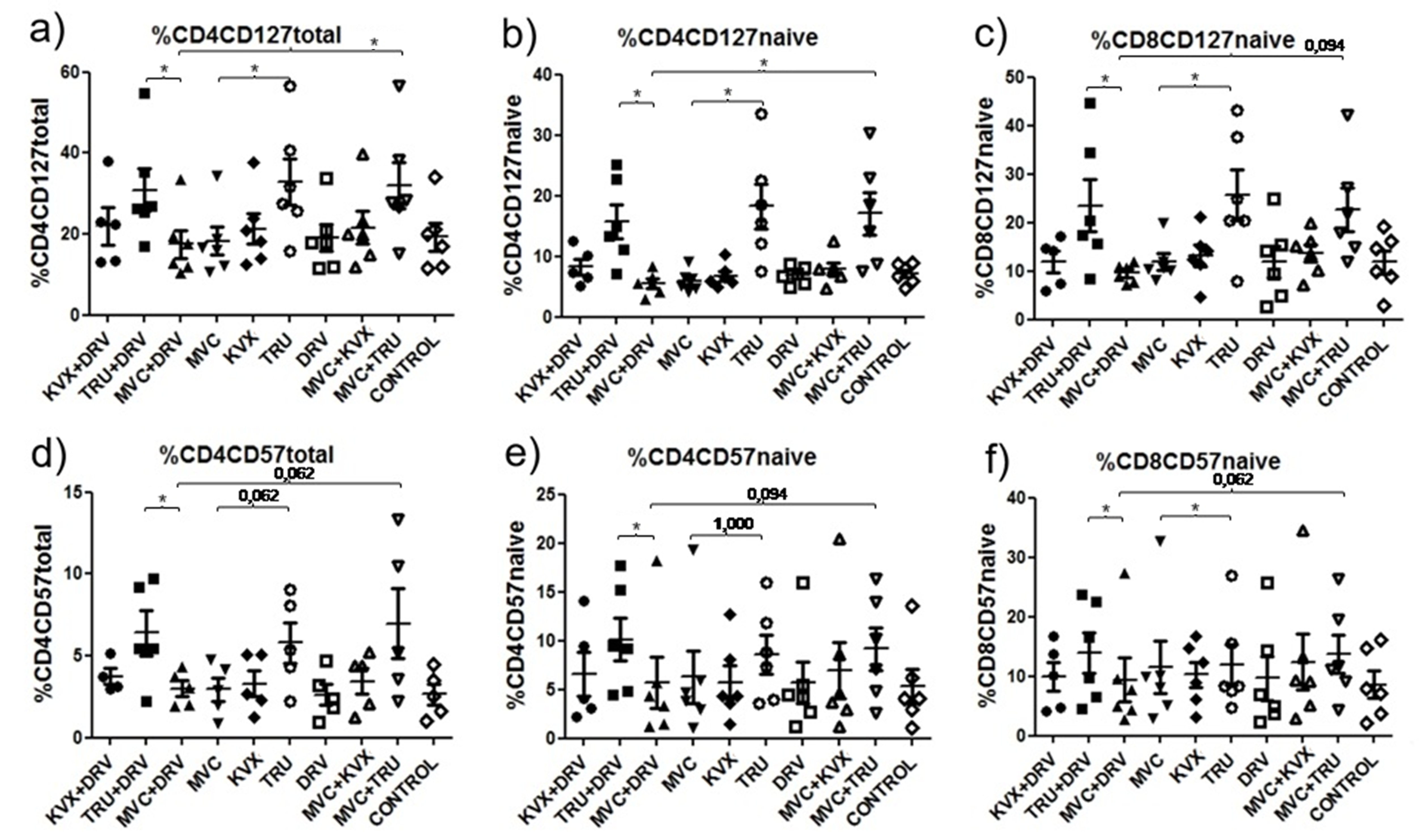


**a-f)** In vitro assays confirmed that the deleterious effect of a NRTIs-containing regimen was due to NRTIs, especially the combination tenofovir plus emtricitabine (TRU, Truvada). KVX, Kivexa (combination of Lamivudine plus Abacavir); DRV, Darunavir; MVC, Maraviroc; CONTROL, No drugs; * Variables with a p-value of <0.05 were considered statistically significant.

**Table S1.** Factors associated with CD8+CD127+ levels after 48 weeks of suppressive cART

| **Patient charasteristics (n=42)** | **Unadjusted OR (95% CI)** ^a^ | **p-value** | **Adjusted OR (95% CI)** | ***p-value*** |
| --- | --- | --- | --- | --- |
| Sex (Male, IQR) | -10.1 [-22.1-1.7] | **0.091** |  | *0.121* |
| Age (years, IQR) | -0.046 [-0.518-0.427] | 0.844 |  |  |
| Time from diagnosis (months, IQR) | 0.129 [-0.041-0.299] | 0.130 |  |  |
| T CD4+ (cells/mm3, IQR) | 0.022 [0.005-0.04] | **0.016** |  | *0.150* |
| Nadir (cells/mm3, IQR) | 0.027 [0.002-0.052] | **0.035** |  | *0.368* |
| Log pVL (copies/mL, IQR) | -4.02 [-8.98-0.89] | 0.104 |  |  |
| T CD8+ (cells/mm3, IQR) | 0.00 [-0.01-0.01] | 0.974 |  |  |
| NRTI-cART | -10.6 [-17.5-(-3.7)] | **0.004** | -10.6 [-17.5-(-3.7)] | ***0.004*** |

Bivariate and multivariate analyses were performed using a linear regression model, and variables showing a p-value of <0.1 (boldface) in the bivariate analysis were included in the stepwise multivariate analysis. In the multivariate analysis, variables with a p-value of <0.05 (boldface) were considered statistically significant. a OR, odds ratio; CI, confidence interval.

**Table S2.** Factors associated with TEM CD4+CD127+ levels after 48 weeks of suppressive cART

| **Patient charasteristics (n=42)** | **Unadjusted OR (95% CI)** ^a^ | **p-value** | **Adjusted OR (95% CI)** | ***p-value*** |
| --- | --- | --- | --- | --- |
| Sex (Male, IQR) | -5.4 [-23.3-12.6] | 0.542 |  |  |
| Age (years, IQR) | 0.167 [-0.501-0.836] | 0.610 |  |  |
| Time from diagnosis (months, IQR) | 0.009 [-0.246-0.263] | 0.945 |  |  |
| T CD4+ (cells/mm3, IQR) | 0.011 [-0.17-0.039] | 0.427 |  |  |
| Nadir (cells/mm3, IQR) | 0.027 [-0.01-0.064] | **0.143** |  | *0.847* |
| Log pVL (copies/mL, IQR) | -0.58 [-12.21-2.05] | **0.154** |  | *0.372* |
| T CD8+ (cells/mm3, IQR) | 0.002 [-0.014-0.018] | 0.824 |  |  |
| NRTI-cART | -14.2 [-24.2-(-4.1)] | **0.008** | -14.2 [-24.2-(-4.1)] | ***0.008*** |

Bivariate and multivariate analyses were performed using a linear regression model, and variables showing a p-value of <0.2 (boldface) in the bivariate analysis were included in the stepwise multivariate analysis. In the multivariate analysis, variables with a p-value of <0.05 (boldface) were considered statistically significant. a OR, odds ratio; CI, confidence interval.

**Table S3.** Factors associated with TEM CD4+CD57+ levels after 48 weeks of suppressive cART

| **Patient charasteristics (n=42)** | **Unadjusted OR (95% CI)** ^a^ | ***p-value*** |
| --- | --- | --- |
| Sex (Male, IQR) | 4.8 [-14.2-23-7] | *0.609* |
| Age (years, IQR) | 0.101 [-0.607-0.809] | *0.771* |
| Time from diagnosis (months, IQR) | 0.0136 [-0.126-0.398] | *0.293* |
| T CD4+ (cells/mm3, IQR) | 0.007 [-0.023-0.038] | *0.610* |
| Nadir (cells/mm3, IQR) | 0.003 [-0.038-0.045] | *0.862* |
| Log pVL (copies/mL, IQR) | -3.36 [-11.08-4.37] | *0.378* |
| T CD8+ (cells/mm3, IQR) | 0.003 [-0.013-0.02] | *0.675* |
| NRTI-cART | 10.8 [-0.6-22.3] | *0.063* |

Bivariate analyses were performed using a logistic regression model. a OR, odds ratio; CI, confidence interval.

**Table S4.** Factors associated with total CD8+CD57+ levels after 48 weeks of suppressive cART

| **Patient charasteristics (n=42)** | **Unadjusted OR (95% CI)** ^a^ | **p-value** | **Adjusted OR (95% CI)** | ***p-value*** | |
| --- | --- | --- | --- | --- | --- |
| Sex (Male, IQR) | 17.4 [(-3.9)-38.6] | **0.105** |  | *0.308* |  |
| Age (years, IQR) | 0.531 [(-0.275)-0.186] | **0.186** |  | *0.164* |  |
| Time from diagnosis (months, IQR) | -0.110 [(-0.424)-0.204] | 0.476 |  |  |  |
| T CD4+ (cells/mm3, IQR) | -0.022 [(-0.056)-0.013] | 0.208 |  |  |  |
| Nadir (cells/mm3, IQR) | -0.028 [(-0.075)-0.019] | 0.237 |  |  |  |
| Log pVL (copies/mL, IQR) | 3.1 [(-6.1)-12.4] | 0.485 |  |  |  |
| T CD8+ (cells/mm3, IQR) | 0.010 [(-0.009)-0.03] | 0.290 |  |  |  |
| NRTI-cART | 17.7 [0.5-30.2] | **0.008** | 17.7 [0.5-30.2] | ***0.008*** |  |

Bivariate and multivariate analyses were performed using a logistic regression model, and variables showing a p-value of <0.2 (boldface) in the bivariate analysis were included in the stepwise multivariate analysis. In the multivariate analysis, variables with a P value of <0.05 (boldface) were considered statistically significant. a OR, odds ratio; CI, confidence interval.
